# Sarcopenia and sarcopenic obesity among community-dwelling Peruvian adults: A cross-sectional study

**DOI:** 10.1371/journal.pone.0300224

**Published:** 2024-04-09

**Authors:** Oscar Flores-Flores, Alejandro Zevallos-Morales, Suzanne L. Pollard, William Checkley, Trishul Siddharthan, John R. Hurst, Antonio Bernabé-Ortiz, Fernando M. Runzer-Colmenares, Miles D. Witham, Jose F. Parodi

**Affiliations:** 1 Universidad de San Martin de Porres, Facultad de Medicina Humana, Centro de Investigación del Envejecimiento (CIEN), Lima, Peru; 2 Asociación Benéfica PRISMA, Lima, Peru; 3 Division of Pulmonary and Critical Care, School of Medicine, Johns Hopkins University, Baltimore, Maryland, United States of America; 4 Center for Global Non-Communicable Disease Research and Training, School of Medicine, Johns Hopkins University, Baltimore, Maryland, United States of America; 5 Division of Pulmonary and Critical Care, Miller School of Medicine, University of Miami, Miami, Florida, United States of America; 6 UCL Respiratory, University College London, London, United Kingdom; 7 Universidad Científica del Sur, Facultad de Ciencias de la Salud, Lima, Peru; 8 NIHR Newcastle Biomedical Research Centre, Newcastle University, Newcastle upon Tyne, United Kingdom; Fondazione Don Carlo Gnocchi, ITALY

## Abstract

**Introduction:**

Sarcopenia and sarcopenic obesity (SO) have emerged as significant contributors to negative health outcomes in the past decade. We aimed to estimate the prevalence of probable sarcopenia, sarcopenia, and SO in a community-dwelling population of 1151 adults aged ≥55 years in Lima, Peru.

**Methods:**

This cross-sectional study was conducted between 2018 and 2020. Sarcopenia was defined as the presence of low muscle strength (LMS) and low muscle mass (LMM) according to European (EWGSOP2), US (FNIH) and Asian (AWGS2) guidelines. We measured muscle strength by maximum handgrip strength and muscle mass using bioelectrical impedance analyzer. SO was defined as a body mass index ≥ 30 kg/m^2^ and sarcopenia.

**Results:**

The study participants had a mean age of 66.2 years (SD 7.1), age range between 60 to 92 years old, of which 621 (53.9%) were men. Among the sample, 41.7% were classified as obese (BMI ≥30.0 kg/m²). The prevalence of probable sarcopenia was estimated to be 22.7% (95%CI: 20.3–25.1) using the EWGSOP2 criteria and 27.8% (95%CI: 25.2–30.4) using the AWGS2 criteria. Sarcopenia prevalence, assessed using skeletal muscle index (SMI), was 5.7% (95%CI: 4.4–7.1) according to EWGSOP2 and 8.3% (95%CI: 6.7–9.9) using AWGS2 criteria. The prevalence of sarcopenia based on the FNIH criteria was 18.1% (95%CI: 15.8–20.3). The prevalence of SO, considering different sarcopenia definitions, ranged from 0.8% (95%CI: 0.3–1.3) to 5.0% (95%CI: 3.8–6.3).

**Conclusion:**

Our findings reveal substantial variation in the prevalence of sarcopenia and SO, underscoring the necessity for context-specific cut-off values. Although the prevalence of SO was relatively low, this result may be underestimated. Furthermore, the consistently high proportion of probable sarcopenia and sarcopenia point to a substantial public health burden.

## Introduction

Sarcopenia is a complex syndrome defined as the pathological decrease of muscle quantity and quality [1, 2]. Sarcopenia is associated with several adverse health outcomes including falls, disability, and death [3, 4]. Similar negative impacts are associated with obesity, the prevalence of which has increased, particularly in low-middle income settings, posing social, economic, and healthcare challenges [5].

Obesity and sarcopenia are closely linked and might interact both pathologically and functionally. Obesity can independently lead to loss of muscle mass and function, due to metabolic derangements, sedentarism, and high co-occurrence of non-communicable diseases [6]. On the other hand, sarcopenia might promote fat accumulation due to reduced total energy expenditure [6]. Unfortunately, assessment of both sarcopenia and sarcopenic obesity (SO) has methodological challenges. For sarcopenia, several working international groups [7, 8] have developed guidelines such as the European Working Group on Sarcopenia in Older People (EWGSOP2), the Foundation for the National Institutes of Health (FNIH), and the Asian Working Group for Sarcopenia (AWGS2) [4, 8, 9]. These guidelines focus on three main aspects: low muscle strength (LMS), low muscle mass (LMM), and low muscle performance (LMP) to classify older adults as sarcopenic. In the case of SO, there is still an ongoing development of a consensus. Studies have used various definitions [10–12], the European Society for Clinical Nutrition and Metabolism has recently proposed defining SO as the coexistence of excess adiposity and LMS [6].

Latin America has one of the highest growth rates for older adults [13], with a faster rise in obesity prevalence than the rest of the world [14]. Unfortunately, there are limited estimates of the prevalence of sarcopenia and SO in this population. Studies that included application of the European (EWGSOP2) cut-offs for muscle strength demand careful consideration of different morphological and nutritional aspects that might influence muscle mass and strength [15–18]. Further, they included formulas for the calculation of muscle mass validated only in Caucasian populations [19], or used indicators of muscle mass that are no longer recommended [20, 21].

Our aim was to estimate the prevalence of probable sarcopenia (low muscle strength), sarcopenia and sarcopenic obesity in a representative community-based sample of adults aged 55 years and older from Lima, Peru. Due to the methodological challenges in defining these conditions, we compared sarcopenia prevalence from three well-established guidelines: EWGSOP2, FNIH and AWGS2. Given the low-resource nature of our community, we anticipated a higher prevalence of both conditions compared to other settings. To enable useful and fair comparisons with similar settings, we also evaluated the prevalence of probable sarcopenia and sarcopenia in selected Latin American countries, applying the same criteria and definition of sarcopenia as in our sample.

## Methods

### Study design and setting

This was a cross-sectional study nested in a large multi-national community-based project called the Global Excellence in Chronic Obstructive Pulmonary Disease Outcomes (GECo) study [22]. At the Lima-Peru site, GECo enrolled an age and sex-stratified random community sample of 3,551 individuals aged 40 years and above, from two urban low resource settings of Lima. Data collection in Lima site started 15th November 2018 and finished 10th February 2020.

The detailed inclusion and exclusion criteria for the GECo study are documented in another publication [23]. Briefly, participants were excluded for the following reasons: self-reported pregnancy; active pulmonary tuberculosis or recent treatment for it; inability to perform spirometry due to recent eye, thoracic, or abdominal surgery, or myocardial infarction within the three months prior to the study visit; or a blood pressure reading exceeding 180/100 mmHg.

The study excluded those who were unable to perform a spirometry for any other reason. The presence of chronic conditions was not a disqualifying factor.

### Study sample and participants

**Fig 1:**
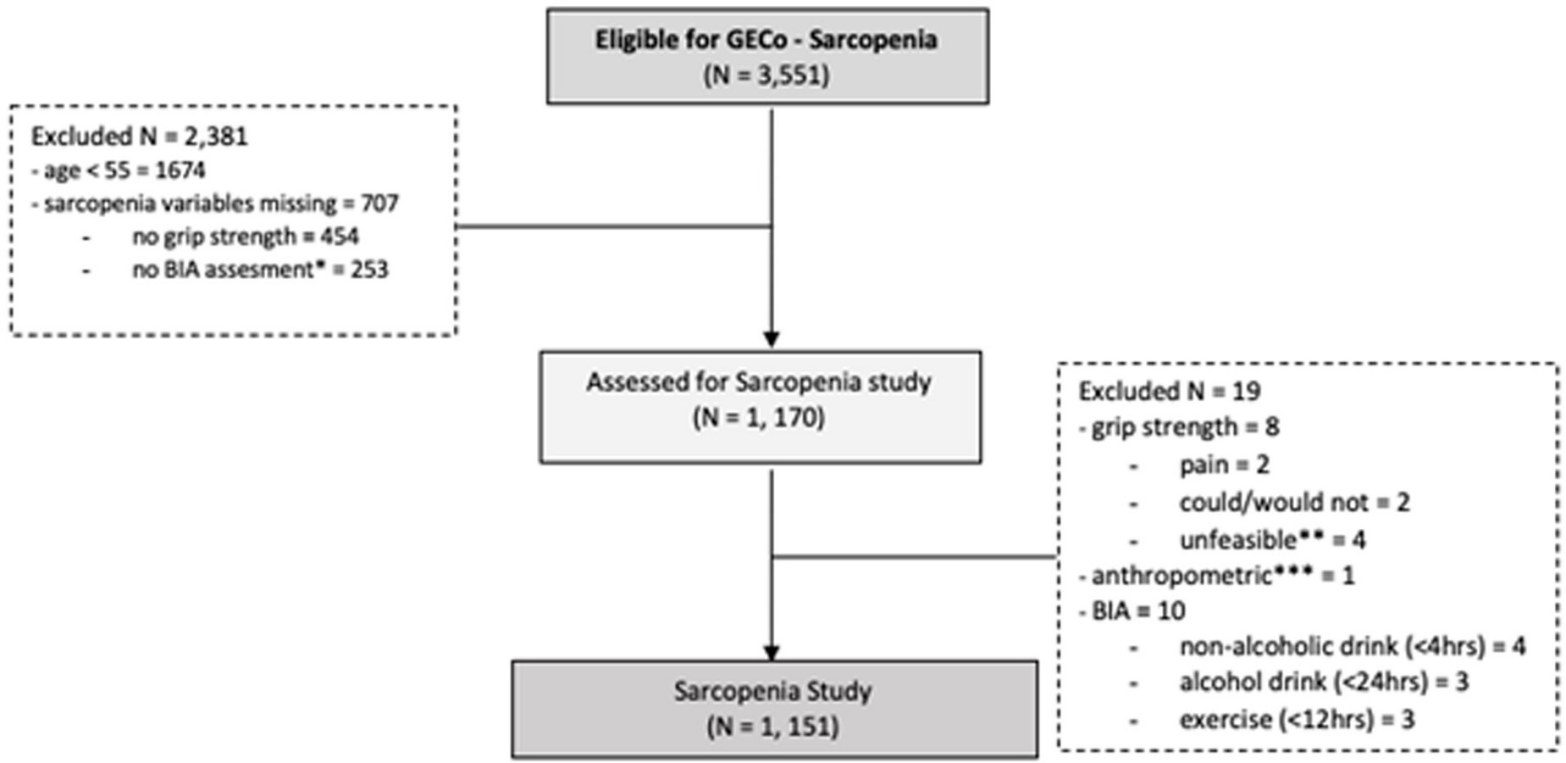
Flowchart of study participants.

For the present analysis, we selected a subset of participants who were ≥ 55 years old, performed handgrip strength testing and underwent bioelectrical impedance analysis (BIA). **Fig 1** shows a flow diagram of the enrolment of the participants.

### Variable measurements

#### Muscle strength

To measure muscle strength, participants performed three handgrip strength trials using a Jamar hydraulic dynamometer while sitting down [*S1 File*]. To define LMS the best trial had to be lower than the cut-off points from these definitions: EWGSOP2 (<27 kg for men; <16 kg for women), FNIH (<26 kg for men; <16 kg for women) and AWGS2 (<28 kg for men; <18 kg for women). The best trial was used since it is less likely to be affected by the number of trials compared to the mean of the trials [24].

#### Muscle mass

Appendicular skeletal muscle mass (ASMM, kg), i.e., the sum of the lean muscle mass of the upper and lower extremities [25], was estimated using the whole-body single-frequency bioelectrical impedance analyzer BodyStat®500 (Bodystat LTD, Douglas, Isle of Man, UK) with the participant in supine position. The resistance (R) at 50 kHz was obtained and ASMM was calculated using the following formula for a non-Caucasian older population [26]:

$$\text{ASMM}\;\text{(kg)}=-0.05376+\left(0.2394*\frac{{\text{H}}^{2}}{\text{R}}\right)+\text{(}2.708*\text{sex)}+\text{(}0.\text{065}*\text{W)}$$

where height (H) is measured in centimeters; BIA resistance (R) is measured in ohms (Ω), and weight (W) is measured in kg; for sex, men = 1 and women = 0. Additionally, we calculated Skeletal Muscle mass Index (SMI) as ASMM/height^2^.

To define LMM, we used 1) the EWGSOP2 criteria for ASMM (ASMM <20 kg & <15 kg), and SMI values (<7.0 kg/m^2^ & <5.5 kg/m^2^), for men & women, respectively; 2) the FNIH criteria using reference for ASMM (ASMM <19.75 kg & <15.02 kg), and ASMM adjusted by the body mass index (BMI) values (ASMM/BMI <0.789 & <0.512), for men & women, respectively, and 3) the AWGS2 criteria using reference for SMI values (<7.0 kg/m^2^ & <5.7 kg/m^2^), for men and women, respectively.

#### Physical performance

To evaluate physical performance, we measured the Short Physical Performance Battery (SPPB) score. The SPPB is based on three timed tasks: standing balance, 4-meter gait speed, and chair stand tests [27]. The timed results of each subtest are scored according to predefined cut-points for obtaining a global score ranging from 0 (worst performance) to 12 points (best performance). Gait speed was measured at usual pace at 4-meter length using the mean of two tests.

LMP was defined using the cut-off points for gait speed, SPPB, and chair time. For the EWGSOP2 definition an SPPB score ≤ 8 or a gait speed ≤ 8 was used. In the AWGS2 definition, an SPPB score ≤ 9 or a 5-chair stand test ≥12 seconds was used.

#### Probable sarcopenia, sarcopenia and sarcopenic obesity

Probable sarcopenia was defined as LMS based on EWGSOP2 and AWGS2 guidelines. Sarcopenia was defined as the presence of LMS and LMM according to the EWGSOP2, FNIH and AWGS2 guidelines. Sarcopenic obesity was defined as the presence of sarcopenia (either EWGSOP2 or AWGS2) and obesity by a BMI equal or greater than 30 kg/m2.

#### Other variables

Height was measured three times with a SECA 213® stadiometer, and weight three times with SECA 803® scale, clothed without shoes. BMI (score and categorized by WHO guidelines; BMI ≤ 24.9 as normal, BMI of 25 to 29.9 as overweight, BMI of 30 to 34.9 as class I obesity, and BMI ≥ 35 as class II obesity or more, all measured in kg/m2). We additionally included age, sex, and level of education (number of years on education and classified).

### Data analysis

We performed a descriptive analysis with means and standard deviation for continuous variables and frequencies and proportions for categorical variables. Prevalence estimates of probable sarcopenia, sarcopenia, and SO were calculated for all included definitions (EWGSOP2, FNIH and AWGS2) with their 95% confidence intervals (CI). Missing data was handled by complete case analysis. Excluded participants characteristics is provided in *S1 Table*. Statistical analysis was performed using STATA 17 statistical software (StataCorp LP).

We compared probable sarcopenia of our Peruvian sample with other Latin American countries, matching each study criteria: LMS cut-off, grip strength calculation, age and sex. We conducted a convenient review of literature, searching databases (PubMed and Google Scholar) for articles published in English or Spanish, with search terms “sarcopenia”, “probable sarcopenia”, “dynapenia” performed in Latin American countries. Since EWGSOP2 or AWGS2 were developed in 2019, we mainly focused in studies performed since January 2019. In countries where no EWGSOP2/AWGS2 were used, we admitted EWGSOP1 guidelines using their respective cut-off. We sought publications from the references lists of identified papers. We obtained prevalences of probable sarcopenia by sex, and utilized the same parameters for calculating probable sarcopenia in our sample.

### Ethics

Ethics permissions were obtained from the University College London Research Ethics Committee (UK), the Institutional Review Board from Johns Hopkins University School of Medicine (US) and the Institutional Committee of Ethics in Research from Asociacion Benefica PRISMA (Peru). All participants provided written informed consent.

## Results

### Characteristics of the sample

Our sample included 1151 participants with a mean age of 66.2 years (SD 7.1), age range (60–92 years old), 621 (54.0%) were men, 44.1% were overweight (BMI of 25 to 29.9kg/m2) and 41.7% were obese (BMI ≥ 30.0kg/m2). General characteristics of the sample are presented in **Table 1**.

**Table 1:** General characteristics of the study sample by sex.

Variables	Total (n = 1,151)	Men (n = 621)	Women (n = 530)
**Age years, mean** ± **SD**	66.2 ± 7.1	66.4 ± 7.4	65.9 ± 6.8
**Age category, n (%)**			
55.0–64.9	521 (45.3)	286 (46.1)	235 (44.3)
65.0–74.9	473 (41.1)	232 (37.4)	241 (45.5)
75 or more	157 (13.6)	103 (16.6)	54 (10.2)
**Height in cm, mean** ± **SD**	153.3 ± 8.5	159.1 ± 6.0	146.6 ± 5.6
**Weight in kg, mean** ± **SD**	69.4 ± 11.9	72.7 ± 11.1	65.5 ± 11.6
**BMI, kg/m2, mean** ± **SD**	29.5 ± 4.5	28.7 ± 3.8	30.5 ± 5
**BMI category, n (%)**			
Normal	163 (14.2)	95 (15.3)	68 (12.8)
Overweight	508 (44.1)	317 (51.1)	191 (36.0)
Class I obesity (BMI 30 to < 35)	344 (29.9)	173 (27.9)	171 (32.3)
Class II obesity or more (BMI 35 or more)	136 (11.8)	36 (5.8)	100 (18.9)
**Education in years, mean** ± **SD**	7.7 ± 4.2	8.8 ± 3.8	6.4 ± 4.3
**Educational level, n (%)**			
No education or incomplete primary school	330 (28.7)	118 (19.0)	212 (40.0)
Primary school (Complete)	252 (21.9)	116 (18.7)	136 (25.7)
High school	460 (40.0)	315 (50.7)	145 (27.4)
University or other higher education	109 (9.5)	72 (11.6)	37 (7.0)

SD, standard deviation

“Age category”, “BMI category” and “Educational level” are presented as both counts and percentages. Variables “Age”, “Height”, “Weight”, “BMI”, “Education in years” are described using their mean values along with standard deviations.

### Muscle parameters

In **Table 2,** we show the mean values of the sarcopenia parameters and proportion of individuals with LMS, LMM, and LMP according to different guidelines. The proportion of individuals selected as having LMS using handgrip ranged between 20.2–31.3%. For LMM, as determined by SMI, the proportion ranged from 16.2% to 20.6%. There was a difference in the proportion of individuals with LMM among women when using the EWGSOP2 (17.4%) compared to AWGS2 (27.0%). Using FNIH classification (ASMM/BMI), the proportion of LMM increased substantially to 81.6% (79.2% in men, and 84.3% in women). In terms of LMP, using gait speed and SPPB score thresholds from EWGSOP2 resulted in substantial variation in prevalence (42.2% vs 8.1%, respectively). However, when applying the AWGS2 criteria for SPPB and Chair Time, the prevalence of LMP was more consistent, ranging between 11% and 12.9%, with a notable sex difference (6.8–8.1% in men and 15.9–18.6% in women).

**Table 2:** Parameters of sarcopenia, and proportions of low muscle strength, low muscle mass and muscle performance according to different definitions.

Variables	Total (n = 1,151)	Men (n = 621)	Women (n = 530)
**Sarcopenia parameters, mean ± SD**			
Handgrip strength, kg	26.3 ± 9.4	32.1 ± 8.3	19.6 ± 5.3
Handgrip strength/BMI	0.9 ± 0.4	1.1 ± 0.3	0.7 ± 0.2
Appendicular skeletal muscle mass (ASMM)	17.2 ± 4.4	20.2 ± 3.2	13.6 ± 2.4
Skeletal muscle mass index (SMI)	7.2 ± 1.3	8.0 ± 1.1	6.3 ± 1
ASMM/BMI	0.591 ± 0.2	0.711 ± 0.1	0.451 ± 0.1
SPPB score (n = 1068)	10.9 ± 1.4	11.2 ± 1.2	10.6 ± 1.6
Gait Speed, m/s (n = 1108)	0.8 ± 0.2	0.9 ± 0.2	0.8 ± 0.2
Chair time, sec (n = 1097)	8.8 ± 2.7	8.3 ± 2.5	9.5 ± 2.8
**Low muscle strength (LMS), n (%)**			
EWGSOP2: Max grip*	261 (22.7)	151 (24.3)	110 (20.8)
FNIH: Max grip	232 (20.2)	122 (19.7)	110 (20.8)
AWGS2: Max grip	320 (27.8)	159 (25.6)	161 (30.4)
EWGSOP2: Chair*	37 (3.5)	13 (2.3)	24 (4.9)
FNIH: Grip/BMI	360 (31.3)	191 (30.8)	169 (31.9)
**Low muscle mass (LMM), n (%)**			
EWGSOP2: ASMM	719 (62.5)	310 (49.9)	409 (77.2)
FNIH: ASMM	693 (60.2)	283 (45.6)	410 (77.4)
AWGS2: SMI	237 (20.6)	94 (15.1)	143 (27.0)
EWGSOP2: SMI	186 (16.2)	94 (15.1)	92 (17.4)
FNIH: ASMM/BMI	939 (81.6)	492 (79.2)	447 (84.3)
**Low muscle performance (LMP), n (%)**			
**EWGSOP2**			
Gait speed (N = 1108)	468 (42.2)	209 (34.9)	259 (50.9)
SPPB (N = 1068)	86 (8.1)	34 (5.9)	52 (10.6)
**AWGS2**			
SPPB (N = 1068)	138 (12.9)	47 (8.1)	91 (18.6)
Chair time (N = 1068)	117 (11.0)	39 (6.8)	78 (15.9)

SD, standard deviation; SPPB, Short Physical Performance Battery; EWGSOP2, European Working Group on Sarcopenia in Older People-2; FNIH, Foundation for the National Institutes of Health; AWGS2, Asian Working Group for Sarcopenia-2.

Variables under ’Sarcopenia parameters’ are described using their mean values along with standard deviations. Variables under ‘low muscle strength (LMS)’, ‘low muscle mass (LMS) and ‘low muscle performance (LMP)’ are presented as both counts (n) and percentages (%). *According to EWGSOP2, having LMS (either with grip strength or chair stand up) is considered as probable sarcopenia.

### Probable sarcopenia, sarcopenia and SO prevalence

In **Table 3**, we show the prevalence of probable sarcopenia, sarcopenia and sarcopenic obesity. We found that the prevalence of probable sarcopenia stood at 22.7% (95% CI: 20.3% - 25.1%) under the EWGSOP2 criteria, and 27.8% (95% CI: 25.2% - 30.4%) when applying the AWGS2 definitions. These rates did not significantly differ between men and women. For sarcopenia, the overall prevalence in our sample varied from 5.7% to 18.1%. When considering the EWGSOP2 and AWGS2 classifications that use the SMI, prevalence rates were 5.7% (95% CI: 4.4% - 7.1%) and 8.3% (95% CI: 6.7% - 9.9%), respectively. The prevalence of sarcopenia showed a statistically significant sex difference with the AWGS2 criteria (6.8% in men vs. 10.2% in women, p = 0.036), but this difference was not observed with the EWGSOP2 criteria (6.4% in men vs. 4.9% in women, p = 0.264).

**Table 3:** Prevalence of probable sarcopenia, sarcopenia and sarcopenic obesity according to EWGSOP2, FNIH and AWGS2 classification.

Variables	Total (n = 1,151)	95% CI	Men (n = 621)	95% CI	Women (n = 530)	95% CI
Probable Sarcopenia* **, n (%)**						
**EWGSOP2**						
Max handgrip strength	261 (22.7)	(20.3% - 25.1%)	151 (24.3)	(20.9% - 27.7%)	110 (20.8)	(17.3% - 24.2%)
AWGS2**						
Max handgrip strength	320 (27.8)	(25.2% - 30.4%)	159 (25.6)	(22.2% - 29.0%)	161 (30.4)	(26.4% - 34.3%)
**Sarcopenia, n (%)**						
**EWGSOP2**						
Max handgrip strength + ASMM	188 (16.3)	(14.2% - 18.5%)	97 (15.6)	(12.8% - 18.5%)	91 (17.2)	(13.9% - 20.4%)
Max handgrip strength + SMI	66 (5.7)	(4.4% - 7.1%)	40 (6.4)	(4.5% - 8.4%)	26 (4.9)	(3.1% - 6.8%)
**AWGS2**						
Max handgrip strength + SMI	96 (8.3)	(6.7% - 9.9%)	42 (6.8)	(4.8% - 8.7%)	54 (10.2)	(7.6% - 12.8%)
**FNIH**						
Max handgrip strength + ASMM/BMI	208 (18.1)	(15.8% - 20.3%)	110 (17.7)	(14.7% - 20.7%)	98 (18.5)	(15.2% - 21.8%)
**Sarcopenic Obesity, n (%)**						
**EWGSOP2**						
Sarcopenia (ASMM) + BMI> = 30	58 (5.0)	(3.8% - 6.3%)	18 (2.9)	(1.6% - 4.2%)	40 (7.5)	(5.3% - 9.8%)
Sarcopenia (SMI) + BMI> = 30	9 (0.8)	(0.3% - 1.3%)	6 (1.0)	(0.2% - 1.7%)	3 (0.6)	(-0.1% - 1.2%)
**AWGS2**						
Sarcopenia (SMI) + BMI> = 30	14 (1.2)	(0.6% - 1.9%)	7 (1.1)	(0.3% - 2%)	7 (1.3)	(0.3% - 2.3%)

SPPB, Short Physical Performance Battery; EWGSOP2, European Working Group on Sarcopenia in Older People-2; FNIH, Foundation for the National Institutes of Health; AWGS2, Asian Working Group for Sarcopenia-2. ASMM, appendicular skeletal muscle mass; SMI, Skeletal Muscle Index; BMI, body mass index; SPPB, short physical performance battery. Variables are presented as both counts (n) and percentages (%).

*No FNIH definition for probable sarcopenia

**This definition is based on the description of possible sarcopenia in the primary care or community setting of the AWGS2 guideline

We observed the proportion of SO between 0.8% and 5.0%. When employing the ASMM definition under the EWGSOP2 criteria, a significant sex-based difference emerged: 2.9% of men versus 7.5% of women were affected (p<0.001). However, when the SMI was utilized as the defining parameter in both the AWGS2 and EWGSOP2 criteria, the prevalence of sarcopenic obesity was approximately 1%, with no observed differences between men and women.

### Prevalence comparison in Latin America

**Table 4** offers a detailed comparison of the point prevalence of probable sarcopenia, aligning our study with nine others from Latin America, all using comparable sex-specific handgrip strength cut-off criteria. Among three community-based Brazilian studies, we noticed substantial differences. Our prevalence rate (27.3%) closely mirrored the first two Brazilian studies with 34.4% and 24.5% respectively [28, 29]. However, the second study [30] showed a stark contrast, with our prevalence at 46.9% versus their 13.6%. A critical factor in this discrepancy was probably their high dropout and deceased participant rate, which reduced their final sample size significantly (from 1284 to 549), possibly skewing it towards healthier individuals.

**Table 4:** Comparison of probable sarcopenia prevalence among community older adults of Latin American countries.

Reference	Country	Year	Characteristics		Probable Sarcopenia prevalence selected study n (%)	Total sample size selected study (n)	*Probable Sarcopenia prevalence in our sample n (%)	** Our Sample size (n)
[28]	Brazil	2022	Age: 60 or more	Total	45 (34.4)	132	246 (27.3)	901
[29]	Brazil	2021	Age: 60 or more	Total	316 (24.5)	1290	246 (27.3)	901
[30]	Brazil	2021	Age: 73 or more	Total	72 (13.6)	529	113 (46.9)	241
[17]	Colombia	2020	Age: 60 or more Grip: Mean of attempts	Male	1041 (42.8)	5237	169 (33.9)	499
				Female	1393 (57.2)		139 (34.6)	402
				Total	2434 (46.5)		308 (34.2)	901
[31]	Chile	2022	Age: 65 or more Grip: 27 kg (Men) & 15 kg (Women)	Total	58 (55.2)	105	201 (31.9)	630
[33]	Chile	2021	Age: 60 or more Grip: 27 kg (Men) & 15 kg (Women)	Male	146 (19.3)	2311	143 (28.66)	499
				Female	298 (19.2)		95 (23.63)	402
				Total	444 (19.2)		238 (26.42)	901
[32]	Chile	2017	Age: 60 or more Grip: 27 kg (Male) & 15 kg (Female)	Male	21 (6.6)	1006	143 (28.66)	499
				Female	44 (6.4)		95 (23.63)	402
				Total	65 (6.5)		238 (26.42)	901
[34]	Mexico	2018	Age: 50–89 Grip: 30 kg (Men) & 20 kg (Women)	Male	160 (78.8)	724	207 (33.3)	621
				Female	116 (22.3)		254 (47.9)	530
				Total	276 (38.1)		461 (40.1)	1151
			Age: 50–89 Grip: 29.1 kg (Men) & 18.4 kg (Women)	Male	27 (13.3)		207 (33.3)	621
				Female	66 (12.7)		229 (43.2)	530
				Total	93 (12.8)		436 (37.8)	1151

Variables are presented as both counts (n) and percentages (%). Unless specified, grip strength was measured as best attempt and EWGSOP2 threshold values were used (<27 kg for men; <16 kg for women). EWGSOP2, European Working Group on Sarcopenia in Older People-2.

*The column ’Probable Sarcopenia prevalence in our Sample’ reflects prevalence derived from applying the same grip strength criteria, as used in each cited study, to our sample.

**The column ’Our sample size’ indicates the number of participants from our study who were included after aligning with the age and sex criteria of each selected study."

In the selected Colombian study [17], the prevalence of probable sarcopenia was considerably higher (46.5% in their study compared to 34.2% in ours). A notable methodological difference was their use of the mean of six handgrip attempts (both hands), in contrast to our use of the maximum value of three trials with dominant hand. When examining the Chilean studies [31–33], some adapted their handgrip strength cut-offs to better fit their specific study cohorts. Applying these tailored cut-offs to our sample resulted in a higher prevalence of probable sarcopenia than those reported in the Chilean studies. Finally, in a community-based Mexican study [34], adopting their unique handgrip strength cut-offs led to a prevalence in our study that was broadly similar, though we observed a significant sex-based variation.

## Discussion

In our community-dwelling sample of adults 55 years and over in Lima Peru, at least one in five adults had probable sarcopenia, the prevalence of which varied due to different cut-offs across guidelines (22.7–27.8%). Furthermore, the prevalence of sarcopenia was lower when using SMI as the muscle mass parameter (5.7% in EWGSOP2 and 8.3% in AWGS2) compared to ASMM (18.1% in FNIH, 16.3% in EWGSOP2). Finally, we found a low prevalence of sarcopenic obesity (0.8–5.0%) using the sarcopenia definition EWGSOP2/AWG2 plus high BMI, despite having a large proportion of obese (BMI> = 30) individuals in our sample (41.7%).

A challenge we faced was the absence of local or Latin American guidelines for determining sarcopenia, unlike the European or Asian contexts. To address this heterogeneity, we applied the same grip strength cut-offs as those used in regional studies to ensure a fair comparison. Nonetheless, we found differences that were probably not only attributed to real differences or variations in target populations or sampling methods, but from methodological differences ascertaining hand grip strength.

The lack of consensus is even more pronounced for sarcopenic obesity. Studies regarding SO are even more scarce in Latin America, although one study in Mexico City that used the same diagnostic criteria, reported a SO prevalence of 2.5% [35], similar to our low prevalence in urban Lima. However, it is important to note that previous studies [36, 37] have highlighted that when using confirmed sarcopenia definition from EWGSOP2 in individuals with high BMI, it underestimates the prevalence of SO due low prevalence of ASMM/height in obese and overweight individuals. Thus, this low prevalence of SO should be taken carefully. Unfortunately, we did not have percentage fat mass (FM) as variable to make comparisons with the consensus of the European Society for Clinical Nutrition and Metabolism and the European Association for the Study of Obesity (ESPEN-EASO) operational definition [6].

### Research implications

Our study highlights the challenges that underpin measurement of sarcopenia and SO in a Latin American setting where there is no regional or local guideline and cut points. This is a call for research investment and collaboration to pool data from Latin America, with the need to develop longitudinal studies that allows determination of valid local cut-offs for hand grip and muscle mass criteria (with BIA or other methods such as ultrasound) associated with negative health outcomes, value our heterogeneity due to different levels of urbanicity and populations living at high altitude. We advocate for the establishment of region-specific guidelines and the promotion of awareness among healthcare professionals and policymakers regarding the importance of context-specific approaches to tackle sarcopenia effectively. The second call is for transparency. We included the manuals we used for the procedures of hand grip strength and muscle mass. However, that is not common practice, and creates high variability. For instance, if hand grip was measured standing up or sitting. Furthermore, although the design of the study did not allow us to recommend which guideline should be used for a Latin American population, there is no clear justification to prefer the European (EWGSOP2) over the Asian cut-offs (AWGS2). In Peru, due to several factors including height, Asian countries’ cut-offs may be more appropriate [38]. Additionally, the validation of local cut-off should follow rigorous methodology, and not only using lowest quintile for each parameter. Finally, regarding SO, we believe that other definitions that includes percentage of fat mass might be a better choice than those who use EWGSOP2 sarcopenia definition plus high BMI.

### Strengths and limitations

Our sample was a census-representative sample of community-dwelling adults, which allowed a better approximation of the community prevalence of sarcopenia, although we acknowledge that some potential participants were excluded because they were unable to perform spirometry and these might be at higher risk of sarcopenia. Additionally, our study did not assess other potentially relevant variables such as physical activity, alcohol, or drug use. Including these could have provided a more comprehensive understanding of the sample. A second strength is the effort to measure the parameters of sarcopenia with accurate methods, i.e., muscle strength measured in three attempts using maximum trial and muscle mass by whole-body bioimpedance analysis with valid equipment. BIA that uses two electrodes in the supine position is more accurate compared to those obtained standing or with only one electrode. Furthermore, we used a BIA formula validated in a similar population in Mexico and not the most common Caucasian formulas used in several Latin American papers. Nevertheless, BIA results vary markedly between measurement tools and populations–a given conversion equation is accurate only for a particular combination of measurement tool and population and use of an equation developed in a Mexican population may not provide accurate results in our Peruvian population. Another limitation is that a considerable number of participants did not have sarcopenia measures because the parent study GECo started before the initiation of the sarcopenia measurements. Due to this temporal discrepancy, a subset of participants did not undergo sarcopenia assessments, leading to missing data for this specific aspect of the study. However, the excluded sample had similar characteristics [*S1 Table*]. Some readers might be concerned about the potential bias in our sample due to the high frequency of obesity. However, it is worth noting that similar frequencies have been reported in studies conducted in the same study setting [22, 39]. Finally, we do not do weight analysis to calculate prevalence estimates, which might lead to some inaccuracies in the estimates, although they do not invalidate comparison across guidelines.

## Conclusions

Our study reveals significant variability in the prevalence of probable sarcopenia, sarcopenia, and sarcopenic obesity (SO) among adults aged 55 and older in low-resource urban areas of Lima, Peru. This variation is evident across different guidelines and measurement parameters. The findings underscore the importance of establishing validated local cut-off points for handgrip strength and muscle mass criteria. Although the prevalence of SO was relatively low, this result may be underestimated. Furthermore, the consistently high proportion of probable sarcopenia and sarcopenia point to a substantial public health burden.

## Supporting information

S1 File Procedure of hand grip strength test. (DOCX)

S1 Table General characteristics of excluded participants. (DOCX)
