# Supplementary material for: Sarcopenia and sarcopenic obesity among community-dwelling Peruvian adults: A cross-sectional study
Source: PLoS One. 2024 Apr 9;19(4):e0300224. doi: 10.1371/journal.pone.0300224 (PMC11003669; doi:10.1371/journal.pone.0300224)
Supplement: S1 File — (DOCX) [file pone.0300224.s001.docx]

**Procedure of hand grip strength test**

This procedure is used to measure the grip force of the fist. Assessing this strength has been shown to be a way to predict current and future health. It can reflect the patient's overall health, physical activity, and even aging. It can also predict trauma outcomes at the level of the upper extremities, postoperative outcomes, and as an index of nutritional level.

**Purpose of the manual**

Ensure correct and uniform measurement of grip force.

**Responsibilities**

It is the responsibility of the meter to use this protocol when measuring the patient's grip force. The principal investigator has the responsibility to ensure that the staff working on the study have the appropriate experience to do so.

**To take into account**

The measurement is performed with the Jamar handheld dynamometer.

- The dynamometer has a double-scale reading that shows the isometric force from 0 - 90 kg (0 - 200 lb). The outer sphere records the result in kilograms and the inner sphere in pounds. It has a retention needle that automatically retains the maximum reading until the device is restarted. The dynamometer handle easily adjusts to five grip positions from 35 – 87 mm in 13 mm increments. Always wear the wrist strap to prevent the dynamometer from falling to the ground if it accidentally falls or slips.

- Use the handle position 2 for the dynamometer measurement.

The patient's nails should ideally be short (length less than 1cm). For purposes of better outcome of the evaluation and that is not uncomfortable for the patient.

- The rest time between one measurement and another is 15 seconds.

**Procedure**

1. Wash your hands and explain the process to the patient.
2. Make sure the dynamometer is clean before use.
3. Ask the patient to remove accessories (watches, bracelets, etc.).
4. Record the patient's dominant hand.
5. Demonstrate to the patient how to hold the dynamometer and test it on yourself. Explain how the outer sphere registers (in kilograms) the best result by squeezing as hard as possible.
6. SIT the participant in a chair (without armrests) as comfortably as possible with the back straight and the arms parallel to the body, extended, with the thumbs facing forward.
7. Ask the patient to keep their feet flat on the floor. You should ask the participant to fold up the coarse of his pants so that he can ensure that he keeps his feet flat on the floor and that they do not rise when pressing the dynamometer.
8. Ask him to place his thumb to one side and his fingers on the other side of the handle so that if the position is correct the fingers and thumb should be visible on the same side of the handle of the appliance (figure 1).
9. The dynamometer will be used in position 2 unless the participant's hand is very small in which case position 1 will be used.
10. Let him know that he will feel as if there is no resistance.
11. Check that the red needle is set to 0 before starting.
12. Ask the participant what their dominant hand is, "What hand do you use to grab your fork or eat, or write your name?"
13. Encourage the patient to squeeze as much as possible to achieve the best result until the needle stops rising (about 3 seconds). Use a standard phrase with all patients: **"tighten... stronger, stronger... stop squeezing"**
14. When the needle stops rising, read the measurement (indicated on the outer dial in Kg) and record the result at the nearest 1kg.
15. If the patient's arm moves, elevates the shoulder, or their feet rise off the floor during the measurement, skip that result and repeat the test.
16. Record three measurements for each hand in a way with the dominant hand.
17. The rest time between one measurement and another is 15 seconds.
18. Use the same type of chair for each measurement.
19. Thank the participant for their collaboration.

**Calibration**

The calibration procedure of the Jamar Handheld Dynamometer is carried out in specialized places. The frequency of external calibrations will be specific to each study, so make sure you know when external calibrations should be performed and ensure that, if necessary, another device is available for use while yours is being calibrated. If well cared for, the device should only be calibrated annually. Manufacturers recommend calibrating your device every six months if it is subjected to frequent vibrations or transported in a car. If the instrument has been dropped or you suspect that the calibration is wrong, take your device to a specialized external service.
