# Supplementary material for: Sarcopenia and sarcopenic obesity among community-dwelling Peruvian adults: A cross-sectional study
Source: PLoS One. 2024 Apr 9;19(4):e0300224. doi: 10.1371/journal.pone.0300224 (PMC11003669; doi:10.1371/journal.pone.0300224)
Supplement: S1 Table — (DOCX) [file pone.0300224.s002.docx]

**Table S1- General characteristics of excluded participants**

| **Variables** | **Total (n=726)** | **Men (n=332)** | **Women (n=394)** |
| --- | --- | --- | --- |
| **Age years, mean ± SD** | 64.7 ± 7.7 | 65.2 ± 7.9 | 64.4 ± 7.6 |
| **Age category, n (%)** |  |  |  |
| 55.0 - 64.9 | 390 (54) | 172 (52) | 218 (55) |
| 65.0 - 74.9 | 252 (35) | 117 (35) | 135 (34) |
| 75 or more | 84 (12) | 43 (13) | 41 (10) |
| **Height in cm, mean ± SD** | 152.4 ± 8.7 | 159.3 ± 5.8 | 146.6 ± 6 |
| **Weight in kg, mean ± SD** | 68.9 ± 13.5 | 72.8 ± 12.2 | 65.6 ± 13.7 |
| **BMI, kg/m2** | 29.7 ± 5.4 | 28.6 ± 4.3 | 30.5 ± 6 |
| **BMI, classified (kg/m2)** |  |  |  |
| Normal | 116 (16) | 58 (17) | 58 (15) |
| Overweight | 300 (41) | 155 (47) | 145 (37) |
| Class I obesity (BMI 30 to < 35) | 220 (30) | 94 (28) | 126 (32) |
| Class II obesity or more (BMI 35 or more) | 90 (12) | 25 (8) | 65 (17) |
| **Education in years, mean** ± **SD** | 7.6 ± 4.4 | 6.4 ± 4.5 | 9 ± 3.8 |
| **Education, categorized** |  |  |  |
| No education or incomplete primary school | 228 (31) | 63 (19) | 165 (42) |
| Primary school (Complete) | 188 (26) | 80 (24) | 108 (27) |
| High school | 237 (33) | 142 (43) | 95 (24) |
| University or other higher education | 73 (10) | 47 (14) | 26 (7) |
